# RNA Sequencing on Muscle Biopsies from Exertional Rhabdomyolysis Patients Revealed Down-Regulation of Mitochondrial Function and Enhancement of Extracellular Matrix Composition

**DOI:** 10.3390/genes16080930

**Published:** 2025-08-02

**Authors:** Mingqiang Ren, Luke P. Michaelson, Ognoon Mungunsukh, Peter Bedocs, Liam Friel, Kristen Cofer, Carolyn E. Dartt, Nyamkhishig Sambuughin, Francis G. O’Connor

**Affiliations:** 1 Consortium for Health and Military Performance, Department of Military and Emergency Medicine, F. Edward Hébert School of Medicine, Uniformed Services University of the Health Sciences, Bethesda, MD 20814, USA; 2 Henry M. Jackson Foundation for the Advancement of Military Medicine, Inc., Bethesda, MD 20817, USA; 3 Department of Anesthesiology, F. Edward Hébert School of Medicine, Uniformed Services University of the Health Sciences, Bethesda, MD 20814, USA; 4 Department of Veterans Affairs, Baltimore VA Medical Healthcare System, Baltimore, MD 21201, USA; 5 Department of Anatomy, Physiology and Genetics, F. Edward Hébert School of Medicine, Uniformed Services University of the Health Sciences, Bethesda, MD 20814, USA; 6 Defense & Veterans Center for Integrative Pain Management, F. Edward Hébert School of Medicine, Uniformed Services University of the Health Sciences, Bethesda, MD 20814, USA

**Keywords:** skeletal muscle, exertional rhabdomyolysis, RNA Sequencing, transcriptomics

## Abstract

Background/Objective: Exertional rhabdomyolysis (ER) is primarily driven by mechanical stress on muscles during strenuous or unaccustomed exercise, often exacerbated by environmental factors like heat and dehydration. While the general cellular pathway involving energy depletion and calcium overload is understood in horse ER models, the underlying mechanisms specific to the ER are not universally known within humans. This study aimed to evaluate whether patients with ER exhibited transcriptional signatures that were significantly different from those of healthy individuals. Methods: This study utilized RNA sequencing on skeletal muscle samples from 19 human patients with ER history, collected at a minimum of six months after the most recent ER event, and eight healthy controls to investigate the transcriptomic landscape of ER. To identify any alterations in biological processes between the case and control groups, functional pathway analyses were conducted. Results: Functional pathway enrichment analyses of differentially expressed genes revealed strong suppression of mitochondrial function. This suppression included the “aerobic electron transport chain” and “oxidative phosphorylation” pathways, indicating impaired energy production. Conversely, there was an upregulation of genes associated with adhesion and extracellular matrix-related pathways, indicating active restoration of muscle function in ER cases. Conclusions: The study demonstrated that muscle tissue exhibited signs of suppressed mitochondrial function and increased extracellular matrix development. Both of these facilitate muscle recovery within several months after an ER episode.

## 1. Introduction

Rhabdomyolysis refers to the breakdown of striated muscle fibers and is characterized by the leakage of muscle cell contents, myoglobin, sarcoplasmic proteins, and electrolytes into the extracellular fluid and circulation [1,2]. The most common causes of rhabdomyolysis are crush injuries secondary to trauma, extreme physical exertion, and metabolic myopathies [2,3,4]. Key features include muscle pain and a sudden, transient elevation of serum creatine kinase (CK) levels. Severe rhabdomyolysis is frequently accompanied by increased urinary secretion of myoglobin (myoglobinuria), which can lead to acute kidney injury (AKI) and a potentially life-threatening metabolic crisis [2,4,5]. Exertional Rhabdomyolysis (ER) is a pathologic muscle breakdown associated with strenuous physical activity and compounded by multiple risk factors. These include low fitness levels, high body mass index, ongoing viral illness, and high altitude and temperature. Certain genetic variants can increase susceptibility to ER [3,6]. Additional risk factors include environmental heat, sickle cell trait, obesity, tobacco smoking, and certain medications [1,7,8]. Overall, the fundamental differences between ER and other forms of rhabdomyolysis lie in their causes, triggers, and affected populations. ER is distinctly linked to intense, repetitive, and prolonged physical exercise, particularly among active-duty military personnel and high-performance athletes. Other forms, however, arise from a diverse array of causes, including trauma, infections, metabolic disorders, and drugs, affecting a wider range of demographics and presenting with varied clinical contexts.

Regardless of the specific trigger, the pathophysiology involves a common pathway of muscle cell damage leading to the release of cellular contents and potentially serious complications like AKI. The cellular pathology of ER centers on the disruption of muscle cell integrity, particularly involving ion homeostasis and energy production, as several review papers have summarized [2,9,10,11]. Briefly, strenuous or unaccustomed exercise can cause direct injury to the sarcolemma and/or lead to energy production failure, impairing the function of key ion pumps such as Na^+^/K^+^ATPase and Ca^2+^ATPase. This impairment increases cellular permeability to sodium ions, resulting in a significant influx of calcium (Ca^2+^) into the muscle fibers. The increased intracellular calcium concentration activates calcium-dependent enzymes, including proteases and phospholipases, which initiate the destruction of myofibrillar, cytoskeletal, and membrane proteins. This process leads to muscle fiber necrosis, releasing intracellular contents such as CK, myoglobin, and electrolytes into the extracellular fluid and circulation. The resultant vicious cycle involves sustained muscle contraction due to elevated calcium, further depleting energy reserves and exacerbating muscle damage.

While the above general cellular pathway involving calcium overload and energy depletion is understood to be the mechanism of muscle cell damage, the underlying mechanisms specific to ER are not universally understood. Studies in animal models, such as horses susceptible to recurrent ER, have successfully used transcriptome analysis (RNA-seq or microarray analysis) to reveal altered gene expression in pathways related to calcium regulation, oxidative stress, and mitochondrial function, demonstrating that these molecular derangements can persist even between episodes [11,12]. However, the provided sources do not offer a comprehensive overview of similar large-scale transcriptome research specifically conducted in human ER populations. The genetic landscape of rhabdomyolysis is known to be heterogeneous [6,9], with underlying causes remaining unknown in many cases despite the identification of various associated genes [13]. Therefore, a significant gap exists in applying comprehensive transcriptome analysis to human ER to pinpoint the precise transcriptional signatures associated with different genetic susceptibilities and how they interact with exercise triggers, which is crucial for improving diagnosis and understanding individual risk.

For this research, we performed RNA sequencing to analyze the transcriptomes of skeletal muscle biopsies from 19 patients with ER and eight healthy controls. The results revealed downregulation of genes involved in mitochondrial energy production. Conversely, the upregulation of genes involved in extracellular matrix composition reconstruction suggests impairment in energy metabolism and muscle tissue structure in ER cases.

## 2. Materials and Methods

The study protocol (ANE-80-3397) was approved by the Uniformed Services University (USU) Institutional Review Board. Before enrollment in the study, informed consent was obtained from each patient.

### 2.1. Patient Cohort and Clinical Information

Nineteen patients with documented episodes of ER were referred and evaluated by the caffeine-halothane contracture test (CHCT) at the USU Malignant Hyperthermia (MH) Diagnostic Laboratory (2005–2019). The primary reasons for referral for the CHCT were exercise-induced rhabdomyolysis of unknown etiology. Patients had no personal or family histories of MH, and/or adverse reactions to anesthesia. Patients denied the use of dietary supplements, statins, anabolic steroids, or any other medications. Clinical data included peak CK levels of greater than 5 times the normal upper limit (200 U/L), presenting symptoms during or after rhabdomyolysis events, pathological muscle histology, and altered muscle enzyme analyses.

### 2.2. Muscle Sample Collection

Muscle biopsies were performed at a minimum of six months after the most recent ER event, and exercise was prohibited for at least one week prior to the biopsy. CHCT was performed following the published standardized procedures of the North American Malignant Hyperthermia Group [14]. The CHCT was considered positive if at least one of three muscle strips, exposed to 3% halothane, developed an increase in resting baseline tension of ≥0.7 g, or if one of three muscle strips, exposed to 2 mM or less caffeine, developed a contracture of ≥0.3 g above baseline tension.

### 2.3. Total RNA Extraction and RNA Sequencing

Total RNA was isolated from muscle biopsy samples using TRIzol reagent (Ambion Life Technologies, Austin, TX, USA). Total RNA sample integrity was assessed using automated capillary electrophoresis on a Fragment Analyzer (Agilent, Santa Clara, CA, USA) using the HS RNA Kit (Thermo Fisher Scientific, Waltham, MA, USA). For all samples with RNA quality number (RQN) > 7.0 were used for sequencing. The Illumina® Stranded Total RNA Prep, Ligation with Ribo-Zero Plus Kit (Illumina, San Diego, CA, USA) was used for library preparation according to the manufacturer’s instructions. Sequencing libraries were quantified by real-time PCR using the KAPA Library Quantification Complete kit (Roche, Indianapolis, IN, USA) and assessed for size distribution and absence of free adapters and adapter dimers on a Fragment Analyzer. Sequencing libraries were pooled and sequenced on a Novaseq 6000 (Illumina, San Diego, CA, USA) using a NovaSeq 6000 S1 reagent Kit (200 cycles) with run conditions generating paired-end reads at 150 bp length.

### 2.4. RNA Sequencing Data Analysis

Raw paired-end RNA sequencing reads were aligned to the human reference genome (GRCh38). The raw sequencing data were first analyzed for quality control, followed by removing the rows with no or nearly no information about the amount of gene expression. We performed pre-filtering to keep only rows with a count of at least 5 reads (count) for a minimal number of samples (8 healthy controls) to reduce the analysis bias. We used the *Salmon* abundance quantification method [15] to calculate transcript abundance based on the transcript fasta files downloaded from Homo_sapiens. GRCh38.cdna.all.fa. The differential gene expression between sample groups was quantified and compared using the R package (v4.4.1) of DESeq2 version 1.30 for direct comparison between samples, as described [16]. We used the design formula (~ Group + Gender + Age) that included the ER case/control variable, and then added sex and age as covariates. This ensured that any differences in gene expression between cases and controls were not due to sex or age differences between the groups. The apeglm algorithm for log fold change shrinkage [17] was used to remove the noise and preserve large differences. Significant genes were determined based on the *p*-value (*p* < 0.05). Volcano plots were generated to visualize significance and fold changes in gene expression obtained from RNA-seq data. In the volcano plot, log fold changes in normalized gene expression in the ER case group compared with the control group were used as the x-axis, and −log_10_ Q values were used as the y-axis.

### 2.5. Functional and Pathway Enrichment Analysis

Gene ontology (GO) enrichment analysis was performed using the R package clusterProfiler (v4.4.4) [18]. GO analysis includes the following three criteria: molecular function (MF), cellular component (CC), and biological process (BP). The enrichplot package (1.16.1) was used to draw circles, histograms, and bubble diagrams. An adjusted *p* value (from the Benjamin-Hochberg method) of less than 0.05 was defined as statistically significant. GSEA was performed using the clusterProfiler package. The data were visualized via the clusterProfiler plug-in gseaplot2 (significance was indicated by *p* < 0.05 or q-value < 0.05).

### 2.6. Statistical Analysis

The mean values of the clinical characteristics were compared between the ER case group and the healthy control group using Student’s *t*-test. Statistical analysis for gene enrichment and pathway analysis was embedded in their package, respectively.

## 3. Results

### 3.1. Clinical Characteristics of ER Cases

Demographic and clinical characteristics of patients (n = 19) and healthy controls (n = 8) are summarized in Table 1. Patients in the ER cohort were ethnically diverse and consisted of Caucasians (n = 12), African Americans (n = 6), and unknown ethnicity (n = 1). Patients were predominantly male (89.5%), with a mean age of 29.9 (range of 20–44 years old). The patient demographic reflected the military population, with the majority of patients being male. All patients had documented episodes of rhabdomyolysis triggered by physical exertion. Concomitant exertional heat illness was reported in 2 cases. Severe muscle pain was the most common complaint during and/or after episodes. Recurrent episodes were observed in 13 patients (68.4%), and the remaining 6 had single episodes. Clinical characteristics and peak CK levels were not significantly different between patients with a single episode or recurrent episodes of ER.

Muscle histopathology revealed no significant diagnostic findings. Most observed minor changes were: rare, mild atrophic fibers, mild denervation atrophy, and scattered, rare esterase-positive atrophic fibers. Myophosphorylase, phosphofructokinase, and AMP-deaminase activities, as well as normal distribution of glycogen and lipids, were found in all biopsied muscle by histopathology. Two cases had experienced AKI, and 6 cases (31.6%) experienced myoglobinuria.

### 3.2. Overview of Differentiation Expression Analysis in Skeletal Muscle Expression Profiling

**Figure 1:**
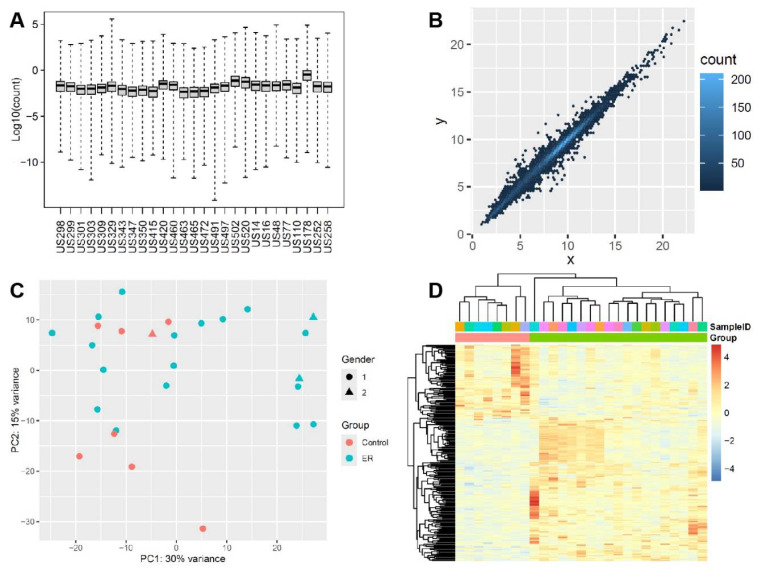
**Analysis overview of differentiation expression profiling in skeletal muscle**. (**A**) boxplot of the Cook’s distances of samples to show if one sample is consistently higher than the others. (**B**) Log2 transformation of the RNAseq count for visualizing/checking any outliers. (**C**) Principal component analysis (PCA) shows the pattern of gene expression profiling between case and control groups. 1 = male and 2 = female. (**D**) Supervised cluster of RNAseq data (padj < 0.1) shows the differential gene expression between two groups.

Transcriptomic profiling using RNA sequencing (RNAseq) allows us to at least indirectly examine how gene expression changes in response to muscle damage from ER. The boxplot of the Cook’s distances (Figure 1A) revealed that the overall gene expression profiles were all within the normal range (no outliers). A total of 15,185 valid gene expression data points were obtained. We transformed these data points using the rlog algorithm for further principal component analysis (PCA) and outlier identification (Figure 1B,C). Supervise cluster analysis (genes with padj < 0.1) clearly showed the difference between the case and healthy control groups (Figure 1D).

### 3.3. Functional Pathway Analysis of Significant Differential Gene Expression

**Figure 2:**
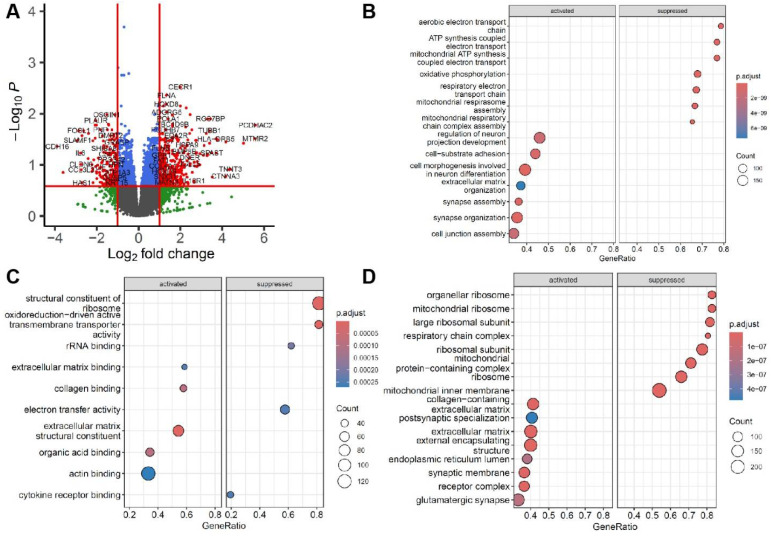
**Functional pathway analysis of significant differential gene expression.** (**A**) The volcano plot shows differential gene expression between the case and control groups. Log_2_ fold change (x-axis), representing the magnitude of gene expression change. Positive values indicate upregulation, while negative values indicate downregulation in the case group. Y-axis: -Log_10_ *p*-value, representing statistical significance. Each dot represents a gene. Bubble plots of functional pathway analysis in different ontology categories show enriched (**B**) biological process (BP), (**C**) molecular function (MF), and (**D**) cellular component (CC) in either activated or suppressed processes. X-axis: GeneRatio (the proportion of differentially expressed genes relative to the total genes in that pathway). Y-axis: Pathway names. Size (gene count): Represents the count of genes in the pathway (larger bubbles = more genes).

Among 15,185 genes detected, 331 genes were significantly upregulated (log_2_FoldChange > 1, *p* < 0.05) and 192 genes were significantly downregulated (log_2_FoldChange < −1, *p* < 0.05) in ER cases compared to healthy controls, as shown in Figure 2A, and the Supplementary Data. Functional pathway analyses of these significantly differentially expressed genes (Figure 2B–D) showed many genes related to mitochondrial function, adhesion, and neuronal development being affected. Stronger enrichment (lower *p* adjust values, higher GeneRatios) in mitochondrial and ribosomal pathways was significantly suppressed in the case group compared with the control group, including “aerobic electron transport chain,” “mitochondrial ATP synthesis coupled electron transport,” and “oxidative phosphorylation” pathways (Figure 2B), in agreement with molecular function (Figure 2C) and cellular component (Figure 2D) ontology analysis. In contrast, ER cases showed enhanced adhesion and extracellular matrix-related pathways compared to healthy controls (Figure 2B–D). Overall, downregulation of mitochondrial energy production and protein synthesis pathways, alongside impacts on neuronal function, were seen in ER cases; however, upregulation of genes involved in adhesion and structural organization was seen in ER cases (Figure 2C,D).

### 3.4. Gene Set Enrichment Analysis of Significant Differential Gene Expression

**Figure 3:**
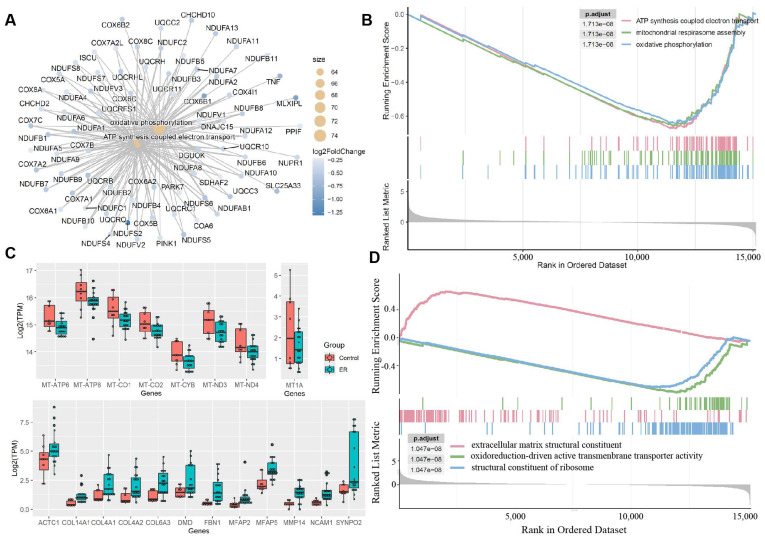
**Gene set enrichment analysis of significant differential gene expression**. (**A**) The R package cnetplot (plot linkages of genes and enriched gene ontology pathways) shows the top 3 biological process pathways with significantly down-regulated genes (blue dots). (**B**) The Gene Set Enrichment Analysis (GSEA) plot displays significant (*p*.adjust < 1.713 × 10^−8^) enrichment scores (y-axis) peaking at the end of the ranked (x-axis) gene list from 3 biological process-related pathways (listed in the panel). (**C**) The boxplot shows expression levels of top genes related to mitochondrial functions and extracellular matrix remodeling between exertional rhabdomyolysis (ER) cases and healthy controls. The y-axis, labeled with Log_2_(TPM), refers to the logarithm base 2 transformation of Transcripts Per Million (TPM) values. (**D**) The GSEA plot presents significant (*p*.adjust < 1.047 × 10^−8^) enrichment scores (y-axis) of 2 molecular function-related pathways (listed in the panel) peaking at the end of the ranked (x-axis) gene, and the extracellular matrix structural constituent network peaking at the beginning of the ranked (x-axis) gene.

Next, to further statistically analyze and visualize which genes are involved in specific enriched functional pathways, we performed the Gene Set Enrichment Analysis (GSEA) using the gseGO function from the R package clusterProfiler [18]. GSEA determines whether a predefined set of genes exhibits statistically significant and consistent differences between ER cases and healthy controls (Supplementary Data). Here, we showed the top 3 pathways impacted by either up- or down-regulated genes in ER cases. We performed GSEA analysis based on the gene ontology (GO) categories of biological process (BP) (Figure 3A–C) and molecular function (Figure 3C,D, Supplementary Figure S1). These analyses revealed significant suppression of mitochondrial pathways, including ATP synthesis coupled with electron transport and oxidative phosphorylation (Figure 3A,B). These pathways are essential for cellular energy production. Meanwhile, enhancement of the extracellular matrix pathway was observed (Figure 3C,D), suggesting activation of skeletal muscle tissue development and reconstruction. Overall, the GSEA results collectively revealed substantial alterations in mitochondrial and extracellular matrix pathways, likely impacting energy metabolism and muscle tissue structure.

## 4. Discussion

Transcriptomic profiling studies on ER can provide insights into its molecular mechanisms. However, several challenges hinder the advancement of transcriptomics research on ER, particularly in humans. First, ER is uncommon, with an incidence of approximately 36.5 per 100,000 patient years in athletes [19] or a similar rate in military service members [20], making it difficult to recruit sufficient participants for large-scale studies. Secondly, obtaining muscle biopsies during acute episodes is invasive, ethically complex, and often impractical, limiting the ability to study gene expression at the critical moment of muscle breakdown. Finally, differentiating between normal exercise-induced gene expression changes and pathological responses in ER is challenging, complicating the interpretation of transcriptomic data. Therefore, research on the transcriptomic landscape of ER has primarily focused on horses [11,12]. While horse studies provide detailed insights, translating these findings to humans is challenging due to differences in muscle physiology and exercise patterns. The current study represents the first transcriptomic comparison and analysis of skeletal muscle tissue between ER patients and healthy controls. Our study results suggested that several genes related to mitochondrial function were down-regulated and genes involved in extracellular matrix structural organization were upregulated in ER cases. We extrapolated that these gene expression changes may be beneficial for repairing muscle tissue after an ER episode. ECM remodeling is essential after muscle injury because the ECM provides structural and biochemical support that facilitates muscle repair and regeneration [21,22,23].

Transcriptome analysis of ER has primarily been conducted in equine models, as described above, particularly in racehorses (like Standardbreds and Thoroughbreds) suffering from recurrent ER. Valberg et al. analyzed gluteal muscle from 9 ER susceptible Standardbreds compared to 7 race-trained controls [11]. Their data indicated genes were enriched in pathways such as inflammation/immune response, cell proliferation, and hypoxia/oxidative stress. Downregulated genes were associated with calcium ion regulation, purine nucleotide metabolism. Consistent with our results (Figure 2B and Figure 3B), their study also revealed inhibition of electron transport in muscle mitochondria.

Research on Thoroughbreds further indicated that ER susceptibility involves alterations in proteins and genes that influence myoplasmic calcium regulation, electron transport, and mitochondrial protein translation [11,12,24], suggesting a fundamental energy deficit and impaired calcium regulation as core mechanisms of ER. However, we did not find significant changes in the genes that regulate the flux of calcium (Ca^2+^) in the muscles. This included upregulation of genes such as RYR1 (ryanodine receptor), calmodulin, calsequestrin, and calpain [11,12,24]. We reasoned that this discrepancy is because our muscle biopsies were sampled at least six months after the most recent ER event, whereas the horse models were biopsied between one and 5.5 h after exercise in the recurrent ER horses. Cellular calcium homeostasis is significantly disrupted during ER due to muscle cell damage, but generally recovers over time following the resolution of the acute episode [9,25].

Overall, our transcriptomic profiling analysis of muscle biopsies from ER patients revealed that suppression of muscle mitochondrial function and activation of extracellular matrix development occurred in skeletal muscle tissues within several months after an ER episode, which may facilitate the restoration of muscle function. This finding may have implications for managing recovery after ER.

## Figures and Tables

**Table 1:** Demographic and Clinical Characteristics of Patients with Exertional Rhabdomyolysis (ER).

Category	ER (n = 19)			Controls (n = 8)		
	Values	Counts	%	Values	Counts	%
Age (year)						
Range	20–44			19–62		
Mean ± SD	29.9 ± 6.7			30.4 ± 14.1		
Gender						
Male		17	89.5		7	87.5
Female		2	10.5		1	12.5
Ethnicity						
African		6	31.6		1	12.5
Caucasian		12	63.2		1	12.5
Unknown		1	5.3		6	87.5
CK level (IU/L)						
Range	2000–170,000			N/A		
Mean	52,272.5			N/A		
Symptoms						
Single episodes		6	31.6		0	
>2 episodes		13	68.4		0	

ER: exertional rhabdomyolysis, SD: standard deviation. N/A: not available.

## Data Availability

All relevant data are within the manuscript and its Supplementary Data. The data at the individual level is not publicly available due to privacy or ethical restrictions.

## Supplementary Materials

The following supporting information can be downloaded at: https://www.mdpi.com/article/10.3390/genes16080930/s1, Figure S1: Gene set enrichment analysis of significant differential gene expression. The cnetplot shows the top 3 Molecular function pathways with significantly up (red dots) or down (blue dots) regulated genes.
