# Supplementary figures and images for: RNA Sequencing on Muscle Biopsies from Exertional Rhabdomyolysis Patients Revealed Down-Regulation of Mitochondrial Function and Enhancement of Extracellular Matrix Composition
Source: Genes (Basel). 2025 Aug 2;16(8):930. doi: 10.3390/genes16080930 (PMC12386086; doi:10.3390/genes16080930)

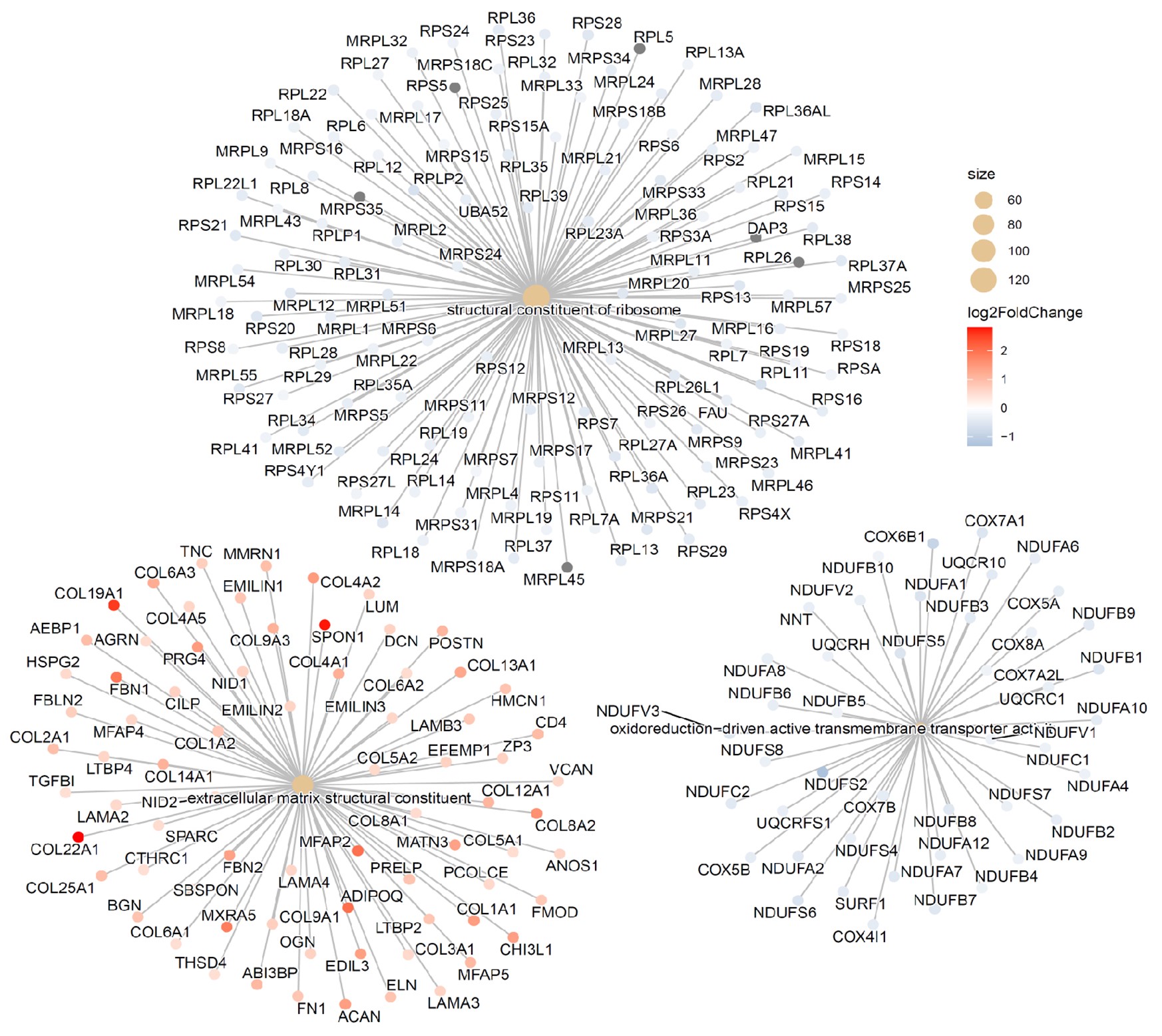

Supplement: Supplementary file 1 [file genes-16-00930-s001.zip › Figure S1 .jpg]
